# Stimulated monocyte IL-6 secretion predicts survival of patients with head and neck squamous cell carcinoma

**DOI:** 10.1186/1471-2407-8-34

**Published:** 2008-01-30

**Authors:** John-Helge Heimdal, Kenneth Kross, Beate Klementsen, Jan Olofsson, Hans Jørgen Aarstad

**Affiliations:** 1 Department of Otolaryngology/Head & Neck Surgery, Haukeland University Hospital, Bergen, Norway; 2 Broegelmann Research Laboratory, University of Bergen, Bergen, Norway

## Abstract

**Background:**

This study was performed in order to determine whether monocyte *in vitro *function is associated with presence, stage and prognosis of head and neck squamous cell carcinoma (HNSCC) disease.

**Methods:**

Prospective study describing outcome, after at least five years observation, of patients treated for HNSCC disease in relation to their monocyte function. Sixty-five patients with newly diagnosed HNSCC and eighteen control patients were studied. Monocyte responsiveness was assessed by measuring levels of monocyte *in vitro *interleukin (IL)-6 and monocyte chemotactic peptide (MCP)-1 secretion after 24 hours of endotoxin stimulation in cultures supplied either with 20% autologous serum (AS) or serum free medium (SFM). Survival, and if relevant, cause of death, was determined at least 5 years following primary diagnosis.

**Results:**

All patients, as a group, had higher *in vitro *monocyte responsiveness in terms of IL-6 (AS) (*t *= 2.03; *p *< 0.05) and MCP-1 (SFM) (*t *= 2.49; *p *< 0.05) compared to controls. Increased *in vitro *monocyte IL-6 endotoxin responsiveness under the SFM condition was associated with decreased survival rate (Hazard ratio (HR) = 2.27; Confidence interval (CI) = 1.05–4.88; *p *< 0.05). The predictive value of monocyte responsiveness, as measured by IL-6, was also retained when adjusted for age, gender and disease stage of patients (HR = 2.67; CI = 1.03–6.92; *p *< 0.05). With respect to MCP-1, low endotoxin-stimulated responsiveness (AS), analysed by Kaplan-Meier method, predicted decreased survival (χ = 4.0; *p *< 0.05).

**Conclusion:**

In HNSCC patients, changed monocyte *in vitro *response to endotoxin, as measured by increased IL-6 (SFM) and decreased MCP-1 (AS) responsiveness, are negative prognostic factors.

## Background

Knowledge about the interactions between tumour cells and the immune system has increased in the last decades. Yet, many basic issues concerning tumour immunology remain unanswered. An intriguing question is why the immune system, capable of eliminating malignant cells under experimental conditions, fails to eliminate tumour cells in patients with progressive cancer disease. Thus, it remains relevant to study functional changes in various immune cells during cancer disease [1,2].

Head and Neck squamous cell carcinoma (HNSCC) is one example of diseases where functional changes in immune cells have been demonstrated [3,4]. Alterations in immune cell functions in HNSCC patients are shown to be both directly disease dependent as well as indirectly related to disease as, e.g., when correlating to impaired general status of patients [5]. Furthermore, it has been shown that *in vitro*-stimulated lymphocyte proliferation, as well as *in vivo *expression of lymphocyte activation epitopes, may be associated with prognosis in HNSCC patients [6,7].

Mononuclear phagocytes (MNPs) are also determined to be functionally changed in patients with HNSCC [8]. Monocytes and macrophages may be separated into type-I and type-II cells according to their cytokine repertoire upon activation [9]. Interleukin (IL)-6 and other pro-inflammatory cytokines are secreted from type-I cells, whereas chemotactic substances such as monocyte chemotactic peptide (MCP)-1 are secreted mainly from type-II cells [9]. When monocytes are stimulated in co-culture with HNSCC tumour cells, high levels of both IL-6 and MCP-1 can be detected in supernatants [10].

IL-6 is a pluripotent cytokine with mostly stimulatory functions. IL-6 may, e.g., act as an autocrine or paracrine growth factor, but also as an anti-apoptotic agent on cancer cells, as is the case in oral cancer [11-13]. MCP-1 was originally determined to recruit macrophages into malignant lesions [14]. MCP-1 receptor expression on tumour cells may be important in the context of tumour cell proliferation and invasion, e.g., in prostate cancer [15].

An increased influx of tumour-associated macrophages (TAMs) in HNSCC tumours is mirrored by a worsened prognosis, but so far no association between monocyte function and survival of HNSCC patients has been published [16]. We have in a previous study observed a correlation between monocyte and TAM IL-6 secretion in HNSCC, suggesting that monocyte function indeed reflects TAM function in HNSCC patients [10]. We therefore suggest that monocyte function may be related to prognosis in HNSCC patients. The aim of the present investigation was to study this hypothesis.

Monocyte function may be assessed by measuring cytokine secretion after *in vitro *stimulation of purified monocytes with endotoxin. We have studied whether monocyte function in HNSCC patients, as measured by *in vitro *endotoxin-stimulated monocyte IL-6 and MCP-1 secretion, was different from monocyte function in control patients and dependent on stage of HNSCC disease as well as prognosis.

## Methods

### Patients and controls

The study comprised patients hospitalised at the Department of Otolaryngology and Head & Neck Surgery, Haukeland University Hospital, Bergen, Norway. Patients had either squamous cell carcinoma (SCC) (N = 65) or benign diseases of the head and neck (HN) (N = 18). Patients with autoimmune disease or patients on corticosteroid medications were excluded from the study. The study was approved by the Regional Committee for Medical Ethics. Each patient gave written consent before participating in the study. Primary sites of carcinomas were: oral cavity (28), pharynx (22), larynx (13), maxilla (1) and unknown primary (1). TNM stages of patients are shown in Table 1. The diagnoses of the patients with benign disease were: dysplastic lesions in the oral cavity or larynx (8), benign tumours in the larynx or middle ear (3), obstructive sleep apnoea (3), benign oesophageal conditions like stenosis or diverticulum (3) as well as tympanic membrane defect (1). Variables such as age, tobacco smoking and alcohol consumption are known to affect monocyte function [17-19]. In order to evaluate the effect of malignant disease per se on immune function, healthy controls, matching cancer patients to these possible confounding variables, were selected. Ages of HNSCC patients (62.2 ± 1.3) and controls (64.4 ± 2.5) were similar. Neither tobacco smoking (33.8 ± 2.5 versus 29.8 ± 5.1 years) nor alcohol consumption history (2.3 ± 0.16 versus 2.1 ± 0.13) differed significantly between the two groups. After at least 5 years post-inclusion, survival was determined from the Norwegian population registry and cause of death from a continuous follow-up registration of HNSCC patients at our department. We found that 30 of the 65 cancer patients were still alive, 27 had succumbed from HNSCC disease and eight from other causes.

**Table 1:** TNM stages of cancer patients evaluated for *in vitro *monocyte function. (Figures represent the final cTNM score of patients before treatment or pTNM scores based on histology from surgery, if such information was present)

	N stage					
	
		0	1	2	3	Sum
T stage	is	2				2
	1	10		6	3	19
	2	12	2	4	1	19
	3	5	2	4		11
	4	8	3	3		14
	Sum	37	7	17	4	65

is = in situ

### Alcohol consumption assessment

Each patient was interviewed at time of inclusion in the study in order to assess smoking and alcohol habits. History of alcohol consumption was scored as follows [20]: 1, no alcohol consumed; 2, modest alcohol consumption (less than once per week); 3, middle level alcohol consumption (1–2 times weekly); 4, alcohol consumed twice weekly; 5, alcohol consumed more than twice weekly.

### Monocyte preparation

Patients were included in the study upon their arrival to the department before any treatment started. All blood samples were drawn at 7.30 a.m. as a bedside procedure and each patient was asked to stay in bed until the blood was drawn. Monocytes were isolated from blood by gradient centrifugation followed by adherence to plastic as previously described. [8] In short, peripheral blood mononuclear cells (PBMC) were separated by gradient centrifugation with Lymphoprep^® ^(Nycomed, Oslo, Norway) as density gradient medium. The PBMC yield of 8.5 ml blood was allocated to a 24-well plate (Nunc A/S, Roskilde, Denmark) with RPMI-1640 (BioWhittaker) supplemented with amphotericin B (2.5 μg/ml) and glucose (both Sigma), HEPES, L-glutamine (2 mM), penicillin (100 IU/ml), streptomycin (100 μg/ml), sodium bicarbonate, sodium pyruvate (all from BioWhittaker) and 20% autologous serum (AS) to a total volume of 0.5 ml/well. After 40 minutes pre-incubation, adherent monocytes were purified by washing, and then cultured in complete RPMI (BioWhittaker)/20% AS with 0.5 ml/well. This method yields more than 95% monocytes positive by non-specific esterase stain with more than 95% viable cells as tested by tryphan blue stain.

### Culture conditions and stimulation

After monocytes were isolated from each donor, cells were without delay further cultured in 0.5 ml/well, either supplied with 20% (AS) or with serum free medium (SFM)(UltraCulture, BioWhittaker). Stimulation was provided for 24 hours by 1 μg/ml lipopolysaccharide (LPS) derived from *Escherichia coli *(Sigma, St. Louis, Mo., USA) before sample collection. Cultures without LPS as stimulant were used as background control conditions.

### IL-6 and MCP-1 analysis

The contents of IL-6 and MCP-1 analysis in supernatants were determined by enzyme-linked immunosorbant assay kit (ELISA) manufactured by R&D Systems (R&D Systems Europe Ltd., Abingdon, Great Britain). All procedures were performed according to the specifications of the manufacturer. Briefly, 96-well microtiter plates (Costar Corning, Lowell, MA, USA) were coated overnight at room temperature (RT) with monoclonal mouse α-human IL-6 or monoclonal mouse α-human MCP-1 capture antibodies. Diluted samples or recombinant human IL-6 or MCP-1 standard were added and incubated for 2 h at RT followed by addition of biotinylated polyclonal goat α-human IL-6 or goat α-human MCP-1. The plates were then incubated for 20 minutes at RT with streptavidin-conjugated horseradish peroxidase. Tetra-methyl-benzidine (TMB) (Sigma) and H_2_O_2 _were used as substrate. Absorbency values were measured at 450 nm using Softmax Pro version 4.0 on an Emax Precision microtiter plate reader (Molecular Devices, Sunnyvale, CA, USA). The lower detection level was 9 pg/ml for IL-6 and 16 pg/ml for MCP-1. The median LPS-stimulated (stimulated – background) IL-6 SFM and AS was 44251 (range 0 – 133014) pg/ml and 40551 (range -8273 – 149411) pg/ml, respectively. The median LPS-stimulated MCP-1 SFM (LPS-stimulated – background) and AS (background – LPS-stimulated) was 7207 (range from -20025 to 66147) pg/ml and -163 (range from -25718 to 13908) pg/ml, respectively.

### Statistical analysis

The statistical program package SPSS (Ver. 14; Inc Chicago, IL, USA) was employed. Figures are given as mean ± standard error of the mean. The groups were compared by the student *t*-test. Survival analyses were performed by Cox regression analysis or Kaplan-Meier (Log Rank (Mantel Cox) test) analyses. When limited versus extended disease patients were compared, the patients were dichotomised by the median of the numeric sum of T and N stage, i.e. the T and N integer score (TANIS) [21] Scores between 0 and 3 were considered as limited disease and scores between 4 and 7 as extended disease. With Kaplan-Meier analyses, monocyte responsiveness was dichotomised into high or low responders by the median value, as measured by the positive account of difference in IL-6 and MCP-1 levels in monocyte cultures when stimulated compared to not stimulated cultures. The median value calculation was based on all included cytokine values. Statistical significance was considered if *P *< 0.05.

## Results

### Monocyte IL-6 and MCP-1 secretion in cancer patients compared to controls

**Figure 1:**
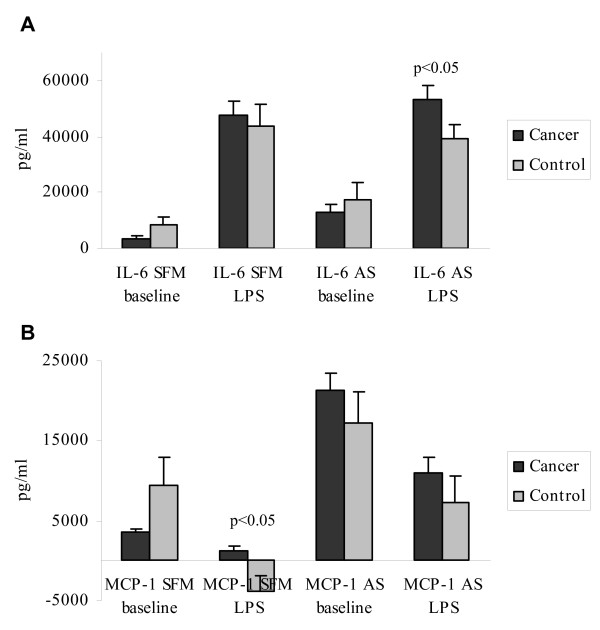
Levels of Interleukin (IL)-6 and Monocyte chemotactic protein (MCP)-1 in supernatants of 24-hours *in vitro *endotoxin (1 μg/ml lipo-poly saccharide (LPS))-stimulated purified monocytes from HNSCC patients and control patients. Cultures were either supplied with autologous serum (AS) or serum free medium (SFM). Bars represent means ± SEM of supernatant levels. (LPS-stimulated – baseline levels: IL-6 SFM/AS & MCP-1 SFM. Baseline – LPS-stimulated: MCP-1 AS). Statistics by students' *t*-test.

When monocytes were cultured with AS and stimulated with LPS, supernatant IL-6 levels in cultures from HNSCC patients were higher compared to those from control patients (53459 ± 4789 pg/ml versus 39165 ± 8179 pg/ml. (*t *= 2.03; *p *< 0.05)). No significant difference could be proven when SFM was utilised (Fig. 1A). A significantly changed endotoxin-induced response compared to background release of MCP-1 secretion from monocytes in cancer patients compared to controls was observed (1232 ± 588 pg/ml versus -3860 ± 1958 pg/ml. (*t *= 2.49; *p *< 0.05)) when cultures were supplied with SFM, but not with AS (Fig. 1B).

### Monocyte IL-6 and MCP-1 secretion as related to disease stage

**Figure 2:**
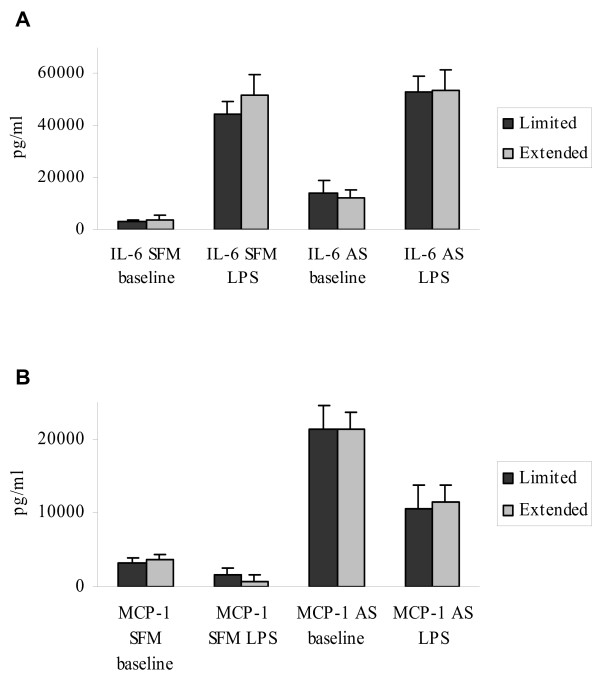
Levels of IL-6 and MCP-1 in the supernatants of 24 hours, *in vitro *endotoxin (1 μg/ml LPS)- stimulated purified monocytes from cancer patients with low (T- + N-stage < 3) (limited) versus high (T- + N-stage > 3) (extended) tumour burden. Cultures were either supplied with 20% autologous serum (AS) or serum free medium (SFM). The bars represent means ± SEM of supernatant levels. (LPS-stimulated – baseline levels: IL-6 SFM/AS & MCP-1 SFM. Baseline – LPS-stimulated: MCP-1 AS).

There were no differences in monocyte endotoxin response, as measured by increased IL-6 secretion (Fig. 2A) or decreased MCP-1 secretion (Fig. 2B), when monocyte cultures from HNSCC patients with extended (TANIS = 4–7) tumour burden were compared to patients with limited (TANIS = 0–3) tumour burden. This held true both with autologous and serum- free medium.

### Prognostic value of monocyte function

**Figure 3:**
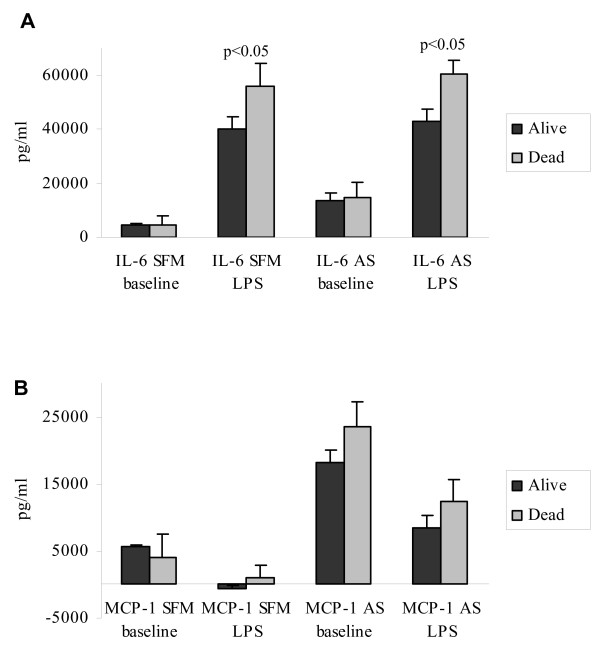
Levels of IL-6 and MCP-1 in the supernatants of 24 hours, *in vitro *endotoxin (1 μg/ml LPS)- stimulated purified monocytes cultures from dead versus live patients with observations at least 5 years following inclusion. Cultures were either supplied with autologous serum (AS) or serum free medium (SFM). The bars represent means ± SEM of supernatant level. (LPS-stimulated – baseline levels: IL-6 SFM/AS & MCP-1 SFM. Baseline – LPS-stimulated: MCP-1 AS). Statistics by students' *t*-test.

With all patients included, endotoxin-stimulated monocyte IL-6 secretion was found to be significantly higher in monocyte cultures from patients that had died after 5 years follow-up compared to that of living patients, both at SFM (*t *= 2.03; *p *< 0.05) and AS (*t *= 2.17; *p *< 0.05) conditions (Fig. 3A). Endotoxin-induced compared to background monocyte MCP-1 secretion was not different in patients alive after five years compared to those that had died during the same period at any of the two culture conditions (Fig. 3B).

**Figure 4:**
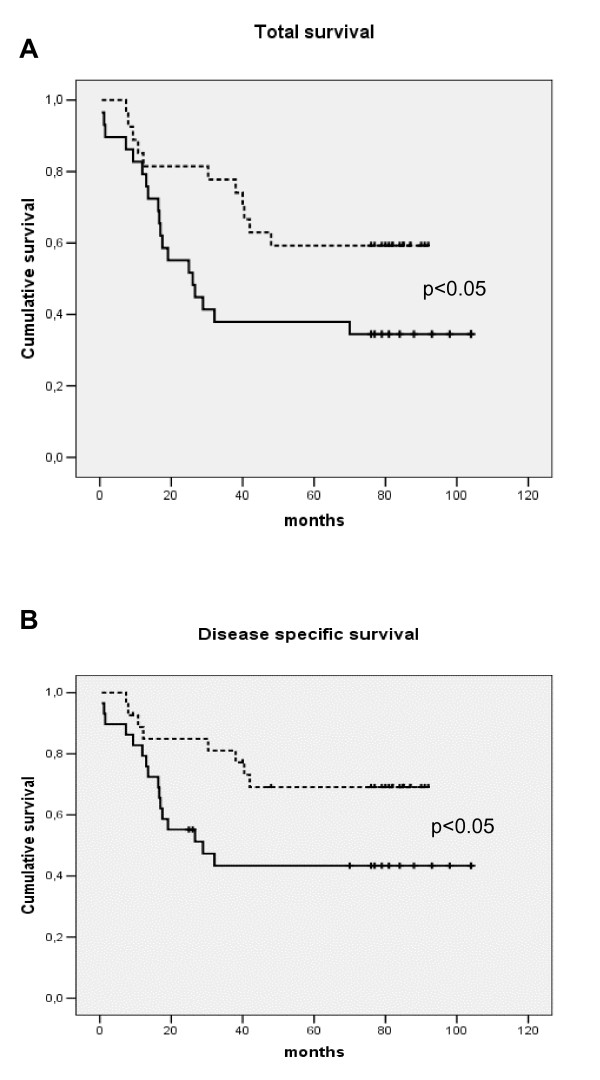
Kaplan-Meier plot survival dependent on total survival (A) or disease-specific survival (B) according to IL-6 *in vitro *secretion from purified monocytes following 24 hours, endotoxin (1 μg/ml LPS) stimulation dichotomised by median value to low (hatched line) or high (continuous line) response with serum free medium (SFM) applied (background subtracted). Statistics by Log Rank (Mantel Cox) test.

**Figure 5:**
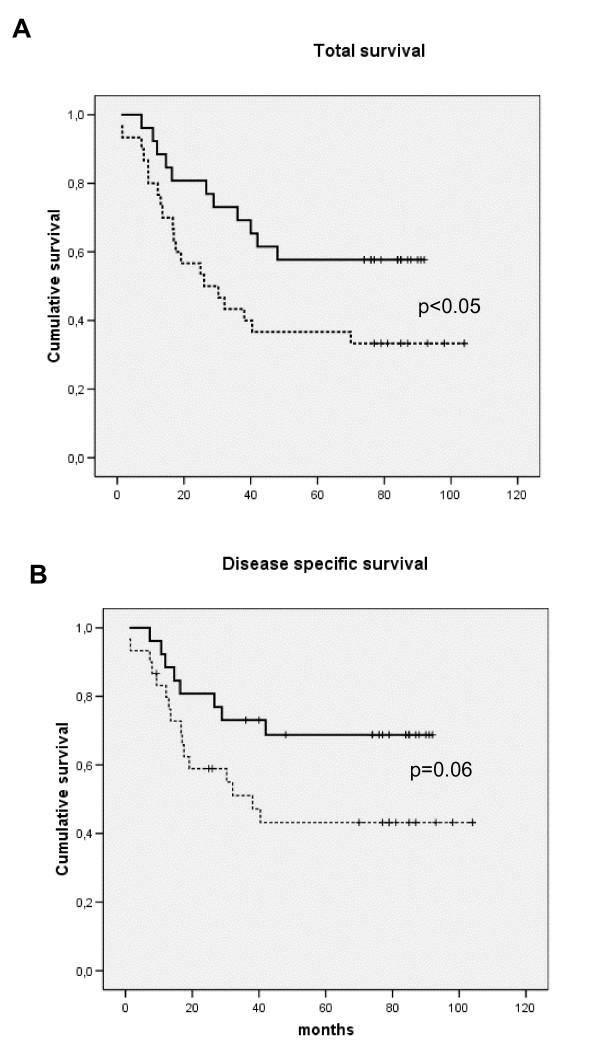
Kaplan-Meier plot survival dependent on total survival (A) or disease-specific survival (B) according to MCP-1 *in vitro *secretion of purified monocytes following 24 hours endotoxin (1 μg/ml LPS) stimulation dichotomised by median value to low (hatched line) or high (continuous line) responsiveness with 20% autologous serum (AS) added to the medium applied (background – LPS stimulated response). Statistics by Log Rank (Mantel Cox) test.

Including HNSCC patients only, Kaplan-Meier analysis showed that patients with high monocyte responsiveness to endotoxin, as measured by high IL-6 secretion (SFM), had decreased total (Fig. 4A) (χ = 4.3; *p *< 0.05) as well as disease-specific (Fig. 4B) (*χ *= 4.4; *p *< 0.05) survival compared to patients with low monocyte responsiveness. When MCP-1 values from AS conditions were analysed by Kaplan-Meier analysis, a low responsiveness predicted decreased total survival (χ = 4.0; *p *< 0.05) (Fig. 5A), and with a trend toward the same with disease-specific survival (χ = 3.6; *p *= 0.06) (Fig. 5B).

With only HNSCC patients included, IL-6 secretion at SFM predicted survival as follows: Cox regression survival analyses, showed that LPS-stimulated monocyte IL-6 secretion, adjusted for gender and age of patients, predicted both total as well as disease-specific survival (*p *< 0.05) when analysed with original results (Table 2, upper panel) and when analysed dichotomised (Hazard ratio (HR) = 2.27; Confidence interval (CI) = 1.05–4.88; *p *< 0.05 and HR = 2.68; CI = 1.11–6.45;*p *< 0.05, respectively) (Table 3, upper panel).

**Table 2:** Multivariate Cox Regression Survival Analysis with Monocyte IL-6 secretion in pg/ml (serum-free medium) Adjusted for Age, Gender and TN stage of HNSCC Patients

	Total survival			Disease-specific survival		
		
	B	SE	*P *value	B	SE	*P *value
	
Gender	-.365	.531	.492	-.707	.654	.280
Age	.062	.021	.003	.068	.024	.004
**IL-6**	**.000**	**.000**	**.029**	**.000**	**.000**	**.024**
						
Gender	-.451	.510	0.779	-.726	.632	0.250
Age	.057	.022	0.022	.055	.025	0.028
T stage	.496	.167	0.003	.647	.195	0.001
N stage	.384	.199	0.054	.420	.230	0.068
**IL-6**	**.000**	**.000**	**0.110**	**.000**	**.000**	**0.066**

B = regression coefficient, SE = Standard error of B

**Table 3:** Multivariate Cox Regression Survival Analysis with Dichotomised Scored Monocyte IL-6 Secretion (serum-free medium) Adjusted for Gender and Age (upper panel), or adjusted for Gender, Age and TN stage (lower panel) of HNSCC Patients

	Total survival			Disease-specific survival		
		
	HR	95% CI (HR)	*P *value	HR	95% CI (HR)	*P *value
	
Gender	0.52	0.18–1.52	0.236	0.35	0.10–1.31	0.119
Age	1.07	1.02–1.11	0.002	1.08	1.03–1.28	0.002
**IL-6**	**2.27**	**1.05–4.88**	**0.036**	**2.68**	**1.11–6.45**	**0.028**
						
Gender	0.54	0.20–1.50	0.237	0.40	0.11–1.41	0.153
Age	1.06	1.02–1.11	0.008	1.06	1.01–1.11	0.025
T stage	1.64	1.18–2.29	0.003	1.89	1.29–2.79	0.001
N stage	1.55	1.06–2.28	0.025	1.61	1.03–2.51	0.035
**IL-6**	**2.08**	**0.97–4.48**	**0.061**	**2.44**	**1.01–5.92**	**0.049**

HR = Hazard ratio 95% CI(HR) = 95% confidence interval for HR

When adjusting for age, gender as well as TNM stage, a prediction for disease-specific survival was determined when IL-6 levels were analysed dichotomised (HR = 2.44; CI = 1.01–5.92; *p *< 0.05) (Table 3, lower panel). A trend towards disease-specific survival prediction was also observed when IL-6 levels were analysed with original results (p = 0.066), (Table 2, lower panel). Likewise, a trend towards survival prediction was observed as to total survival when IL-6 levels were analysed dichotomised (HR = 2.08; CI = 0.97–4.48; p = 0.061), (Table 3, lower panel). Furthermore, when monocyte LPS-stimulated IL-6 secretion values were analysed dichotomised, survival prediction was also present with disease-specific survival (HR = 2.62; CI = 1.14–6.08; *p *< 0.05) and total survival (HR = 3.10; CI = 1.15–8.39; *p *< 0.05), when adjusted for gender and age as well as serum albumin and erythrocyte sedimentation rate (ESR) values of patients (analyses not shown). When adjustment for TNM stage was additionally introduced, predictions were still observed, both as measured by disease-specific survival (HR = 2.31; CI = 1.02–5.21; p < 0.05) and total survival (HR = 2.67; CI = 1.03–6.92; p < 0.05) (analyses not shown).

When dichotomised MCP-1 values were introduced in a Cox regression survival analysis, adjusted for age and gender of patients, we determined a trend as to prediction of total survival at the AS condition (HR = 1.99; CI = 0.95–4.18; p = 0.069) (Table 4, upper panel). Adjusting for age, gender as well as TNM stage of patients, we determined prediction of survival at SFM conditions (HR = 2.42; CI = 1.03–5.69; p < 0.05), (Table 4, lower panel).

**Table 4:** Multivariate Cox Regression Total Survival Analysis with Dichotomised Scored Monocyte MCP-1 Secretion adjusted for Gender, Age, and TNM stage of HNSCC Patients

	Serum-free medium			Autologous serum medium		
		
	HR	95% CI (HR)	*P *value	HR	95% CI (HR)	*P *value
	
Gender	0.73	0.26–2.03	0.546	0.61	0.24–1.59	0.311
Age	1.05	1.01–1.09	0.014	1.04	1.00–1.08	0.027
MCP-1	1.20	0.58–2.48	0.617	1.99	0.95–4.18	0.069
						
Gender	0.41	0.12–1.41	0.166	0.55	0.22–1.41	0.216
Age	1.05	1.00–1.10	0.043	1.04	1.00–1.09	0.036
T stage	2.01	1.41–2.87	0.000	1.57	1.12–2.20	0.008
N stage	1.84	1.16–2.90	0.009	1.50	1.02–2.22	0.041
MCP-1	2.42	1.03–5.69	**0.043**	1.28	0.57–2.89	0.554

HR = Hazard ratio 95% CI(HR) = 95% confidence interval for HR

## Discussion

In this study, we have examined monocyte responsiveness, as measured by *in vitro *endotoxin responsiveness, by monocyte IL-6 and MCP-1 secretion. Monocyte responsiveness was increased in monocytes from HNSCC patients compared to control conditions. On the other hand, no difference in monocyte responsiveness was found when HNSCC patients with limited versus extended disease were compared. Patients with high monocyte responsiveness as measured by IL-6 secretion at serum-free conditions had lower disease-specific survival than patients with low such monocyte responsiveness. Predictions for survival based on monocyte IL-6 secretion, were still valid after adjusting for gender, age, TNM stage, albumin and ESR levels. Furthermore, we determined to some extent that MCP-1 secretion following endotoxin stimulation was related to prognosis. We have, however, determined a much more thorough correlation to prognosis with IL-6 levels than with MCP-1 levels. We therefore suggest that MCP-1 level survival prediction should be more closely studied before any firm conclusions can be drawn. Our observations are in line with results from a recent study by Clinchy and co-workers showing that increased IL-6 secretion, in short-duration *in vitro *cultures of peripheral blood mononuclear cells stimulated with LPS, was associated with impaired prognosis in patients radically operated for colon cancer [22]. Monocyte IL-6 and MCP-1 secreted from endotoxin- stimulated monocytes may be linked to an altered inflammatory state as previously shown in HNSCC patients. Examples are increased ESR, lowered albumin values in serum, increased levels of acute-phase proteins and pro-inflammatory cytokines [23,24]. This has been studied by adding serum albumin and ESR level information to the Cox regression analyses. We determined only minor explanatory power upon adjusting for ESR and albumin in serum. On the contrary, to some extent the IL-6 secretion level, serum albumin levels and ESR independently predicted survival.

We found no association between tumour burden and monocyte function in the present study. This argues against monocyte function being linearly regulated by HNSCC disease-related factors, such as cytokines secreted from tumour-associated cells. The findings in the present study indicate that monocyte changes are generally present in malignant disease and to a lesser extent influenced by tumour burden.

There is evidence to claim that nuclear factor -κB (NF-κB), which regulates expression of multiple genes in cells, may act as a link between infection, inflammation and carcinogens in development of cancer [25]. The pro-inflammatory cytokine IL-6 may have an important role in this process by serving as an anti-apoptotic agent through activation of NF-κB [26]. Our present finding, that increased IL-6 secretion from monocytes predicts prognosis of HNSCC disease, further supports the notion that inflammatory responses may cause both initiation and progression of neoplastic growth [12].

Another effect of IL-6 is its increased promotion of monocyte differentiation towards macrophages at the expense of dendritic cell (DC) differentiation [27]. Monocytes are recruited by chemokine gradients to migrate from circulation into tumour tissues where a further differentiation to TAMs or DCs takes place under the regulation of environmental signals of such as IL-6 [27]. There is currently an increasing agreement that TAMs in carcinoma disease may support tumour growth by virtue of their differentiation into type II macrophages [28]. Compared to TAMs, DCs apparently have a contrary effect within HNSCC tumours, whereby a high number of DCs correlates with better prognosis [29,30]. Furthermore, it has previously been demonstrated that monocytes maintain IL-6 secretion throughout their differentiation to macrophages when continuously stimulated with HNSCC tumour spheroids *in vitro *[31]. We therefore suggest that the malignancy potential of HNSCC relies to some extent on IL-6 stimulation by TAMs.

MCP-1 regulates TAM influx into tumours and may also be secreted by TAMs [10]. It is therefore possible that MCP-1 mediates a self-enhancing effect driven by TAMs within tumours. Increased expression of MCP-1 in squamous cell carcinomas of the oesophagus has been associated with increased influx of TAMs and an impaired prognosis [32]. *In vitro *experiments indicate that these findings may be relevant for HNSCC as well [16]. To what extent the shown lowered monocyte MCP-1 responsiveness association to increased prognosis can be linked to TAM influx in HNSCC tumours needs to be further elucidated.

The observations in the present study add weight to the arguments that activated MNPs may in fact increase rather than reduce tumour cell aggressiveness in HNSCC. Still, TAMs may in some cytokine environments have tumour suppressive potentials, which probably explains observations of improved prognosis associated with high numbers of TAMs in some other types of malignancies [33,34]. The observed reductions in HNSCC tumour mass when injected with biological response modifiers such as OK-432, may be in part be explained by such macrophage activation [35].

Previously, it has been shown that monocytes in HNSCC patients compared to control patients are primed for an increased sensitivity to endotoxin stimulation as measured by cytokine secretion [8]. The present study confirms these observations and further shows that monocyte function actually may provide information as to prognosis of HNSCC disease. We have previously determined in another patient sample that IL-6 secretion from monocytes did not predict survival. It should, however, be noted that patients with more affected capability, as measured by Karnofsky scores below 75, were included in the present study as opposed to a previous study [20]. Furthermore, when both of these samples were combined, prediction relying on monocyte IL-6 secretion was similar to this study (manuscript in preparation).

Both alcohol consumption and tobacco smoking are expected to be higher among HNSCC patients than in the general population because consumption of these substances has been linked to an increased risk of HNSCC [36]. Smoking and alcohol use may influence monocyte function [18,19,37]. In the present investigation, however, differences between HNSCC patients and control patients, as well as differences between the two HNSCC patient groups, and prognosis were present still after adjusting for tobacco and alcohol consumption. The observed changed monocyte function in HNSCC patients can therefore not be explained by alcohol consumption or tobacco smoking.

Monocyte sensitivity to endotoxin reflects prognosis when adjusted for TNM stage. Therefore it may be possible to identify patients having a better prognosis despite extended HNSCC disease. This might justify the use of a more extensive therapy regime in some selected patients with otherwise very extended TNM stage.

Observations from *in vitro *studies suggest that IL-6 promotes cell proliferation and prevents apoptosis in HNSCC cell lines via activation of signal-transducers-and-activators-of-transcription-3 (STAT3) via a common β-chain of the epidermal growth factor receptor (EGFR) [38]. STAT3 plays a critical role in the oncogenesis of several malignancies and has been shown to be activated in tumour tissue and in normal mucosa of HNSCC patients [39]. The activation of STAT3 is, however, shown to be complexly regulated via different kinases and supressors of cytokine signalling genes, which may explain the failure of treatment protocols based on EGFR- tyrosine kinase inhibitors [40]. Lee and co-workers therefore suggest that multiple pathways to stimulate STAT3 should be targeted in patients with HNSCC in order to achieve maximal clinical efficacy [40]. Thus, one possible additional therapeutic pathway could be an inhibition of the IL-6 stimulation of the tumour cells through therapeutic use of anti-IL-6 antibodies [41].

## Conclusion

We have shown that monocyte function, as measured by endotoxin-induced *in vitro *monocyte regulation of IL-6 secretion at AS conditions was higher, whereas, MCP-1 secretion at SFM condition was less inhibited in HNSCC patients compared to controls. Monocyte function also predicted outcome in HNSCC patients. A high LPS-induced monocyte IL-6 responsiveness, and to some extent decreased MCP-1 responsiveness, predicted worsened prognosis independent of TNM stage. Thus, monocyte function is directly associated with the biology of HNSCC. We suggest that future studies should take into account the possible use of α-IL-6 antibodies as an adjuvant treatment for HNSCC disease.

## Competing interests

The author(s) declare that they have no competing interests.

## Authors' contributions

JHH planned and designed the study together with HJA who also performed the statistical analysis, helped draft the manuscript and critically revised the manuscript. JHH included each patient in the study and wrote the manuscript together with KK, who also took a major part when the manuscript was drafted. BK performed most of the laboratory work and carried out the immunoassays. JO revised the manuscript critically. All authors read and approved the final manuscript.

## Pre-publication history

The pre-publication history for this paper can be accessed here:


